# Enhancing metabolic activity and differentiation potential in adipose mesenchymal stem cells via high-resolution surface-acoustic-wave contactless patterning

**DOI:** 10.1038/s41378-022-00415-w

**Published:** 2022-07-12

**Authors:** Karina Martinez Villegas, Reza Rasouli, Maryam Tabrizian

**Affiliations:** 1 Department of Biological and Biomedical Engineering, McGill University, Montreal, QC Canada; 2 Faculty of Dental Medicine and Oral Health Sciences, McGill University, Montreal, QC Canada

**Keywords:** Engineering, Materials science

## Abstract

Acoustofluidics has shown great potential for label-free bioparticle patterning with excellent biocompatibility. Acoustofluidic patterning enables the induction of cell–cell interactions, which play fundamental roles in organogenesis and tissue development. One of the current challenges in tissue engineering is not only the control of the spatial arrangement of cells but also the preservation of cell patterns over time. In this work, we developed a standing surface acoustic wave-based platform and demonstrated its capability for the well-controlled and rapid cell patterning of adipose-derived mesenchymal stem cells in a high-density homogenous collagen hydrogel. This biocompatible hydrogel is easily UV crosslinked and can be retrieved within 3 min. Acoustic waves successfully guided the cells toward pressure nodal lines, creating a contactless alignment of cells in <5 s in culture media and <1 min in the hydrogel. The acoustically patterned cells in the hydrogel did not show a decrease in cell viability (>90%) 48 h after acoustic induction. Moreover, 45.53% and 30.85% increases in metabolic activity were observed in growth and differentiation media, respectively, on Day 7. On Day 14, a 32.03% change in metabolic activity was observed using growth media, and no significant difference was observed using differentiation media. The alkaline phosphatase activity showed an increase of 80.89% and 24.90% on Days 7 and 14, respectively, for the acoustically patterned cells in the hydrogel. These results confirm the preservation of cellular viability and improved cellular functionality using the proposed high-resolution acoustic patterning technique and introduce unique opportunities for the application of stem cell regenerative patches for the emerging field of tissue engineering.

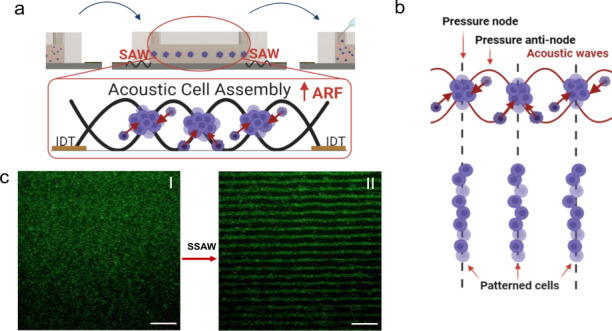

## Introduction

Biological tissues rely on microenvironmental cues, including homotypic and heterotypic cell–cell contacts, extracellular matrix (ECM) stimuli, mechanical forces, and chemical signaling. Cell-to-cell signaling is crucial in numerous biological processes, including the kinetics of aggregation in new tissues, cell migration, proliferation, differentiation, and organogenesis^1–6^. Intercellular communication is also instrumental during cell aggregation and cell assembly processes and is the first step in tissue development. Three processes are often involved in the compaction and formation of cellular patterns, including the interactions between the ECM and integrin as physical linkers for promoting cellular attachment, the upregulation of cadherins upon cell–cell aggregation, and the homophilic interactions of E-cadherins to initiate strong cell adhesion^7,8^. Intercellular adhesion proteins, including connexins and pannexins, also regulate cell–cell interactions and respond to the forces experienced by the cell, which modulate the strength of adhesion and the activity of mechanosensitive signaling pathways and contribute to the formation of cell patterns and organized structures^8,9^. By facilitating cell–cell contacts via controlled spatial arrangements, cell signaling and communication can be enhanced, improving tissue functionality and replicating the native tissue architecture^10^.

Despite the interest in creating complex cellular constructs to mimic organized tissues, fabrication methods for controlling the spatial location of cells face many limitations in terms of efficiency and practicality. Conventional methods for cell patterning, including photolithography stamps^11^, optical tweezers^12^, magnetic patterning^13^, dielectrophoresis^14^, and 3D printing^15^, often involve long fabrication times, cell pattern heterogeneity, lack of cell density control and cell cytotoxicity, require conductive media, have low spatial resolution, and/or lack reproducibility. Furthermore, contact and high energy-based cell patterning methods can also cause alterations in the cell phenotype, which may affect cell viability and functionality.

Acoustofluidics, the combination of acoustics and microfluidics, has been widely explored in the past two decades due to its high spatial and contactless control coupled with its rapid operating mechanism. These characteristics, together with its hydrogel compatibility, allow the preservation of cell patterns and render acoustofluidics an attractive method in tissue engineering for cell spatial patterning. Typically, the first step in tissue engineering using acoustic radiation forces involves the initial organization of cells into a desired pattern. This step is then followed by a preservation step in which the cell pattern is maintained over time in order for cells to establish connections and mature into tissues^16^.

While there are two main acoustic modes for cell manipulation, surface acoustic waves (SAWs) and bulk acoustic waves (BAWs), SAWs offer greater advantages for cell patterning, as they have higher resolution and lower power losses. The high frequency range of SSAWs in the megahertz to gigahertz region allows short wavelengths^17^, which minimizes power consumption, making it a gentle manipulation method^18,19^. Moreover, the micron-order wavelengths in SAWs allow high spatial resolution for the manipulation of single cells, where the operating frequency is confined to a singular frequency and is dictated by the design of interdigitated transducers (IDTs). Due to the piezoelectric effect, an electrical potential results in mechanical vibrations that propagate across the surface of a piezoelectric material and through any secondary material coupled to the SAW device^20^. The configuration and location of the IDTs also determine the type of SAW, which can generate traveling or standing waves. In particular, standing surface acoustic waves (SSAWs) are formed by placing opposite-facing pairs of IDTs on a piezoelectric substrate. Upon the transmission of a radio frequency signal to the pair of IDTs, the opposite-traveling waves create regions of low and high acoustic pressure, namely, pressure nodes and antinodes, respectively. Therefore, cells can be guided toward the pressure nodes to create defined patterns.

Different biomaterials for cell patterning preservation have been proposed to help construct tissue architectures. For instance, 3D fibroblast tissues were formed using NIH 3T3 fibroblasts in a solution of thrombin mixed with fibrinogen at an adjustable concentration, wherein structures were crosslinked in 10 min^21^. Cage-like structures were shown after 30 h due to cell migration, growth, and interactions with F-actin alignment. Conversely, in a collagen type I hydrogel, breast cancer cell line MCF-7 cells were patterned and polymerized after 10 min at neutral pH and 37 °C by heat transfer from the IDTs, thus preserving cell viability^22^. In another study, coaligned HUVECs and hASCs were patterned and preserved in a catechol-conjugated hyaluronic acid scaffold and further implanted in a mouse model to create a functional collateral vascularized cylindroid for ischemia therapy^2^.

One limitation of these biomaterials, however, is the crosslinking time and uniformity of the scaffolds. As a solution, hydrogels that polymerize rapidly, such as photocurable polymers, can combine SSAW rapid patterning and crosslinking within a few minutes. Using different concentrations of PEGDA and GelMA^®^ allowed the rapid crosslinking of HeLa, MC3T3-E1, and P12Adh cells via UV light to form cell-hydrogel patterns in capillary tubes in less than 5 min^23^. Similarly, cardiomyocytes were mixed with a GelMA^®^ solution followed by SSAW-patterning and UV crosslinking, where cardiomyocytes showed beating activity for up to 7 days^3^. Despite the similarity of the working methods for linear SSAW-patterning, the effects of the acoustic patterning of adipose-derived stem cells in a 3D environment for the preservation of cell viability, improvement in metabolic activity, and differentiation of stem cells into an osteogenic lineage as a proof-of-concept of a regenerative tissue patch have not yet been reported to our knowledge.

Therefore, in this work, we propose a SSAW-based cell patterning platform for fabricating rapid cell-laden hydrogel constructs using a methacrylated collagen type I hydrogel (PhotoCol^®^-LAP) with the capability of being retrieved from the platform within 3 min of photo crosslinking. Collagen is the most abundant component of the ECM, and it is commonly used in tissue engineering because it recapitulates the native tissue microenvironment^24^. Furthermore, the flexibility of PhotoCol^®^-LAP as a UV-sensitive hydrogel can preserve the high resolution of SSAWs by rapidly crosslinking the aligned cells. A systematic experimental plan was used to demonstrate that the proposed acoustic force-based microfluidics platform has the capability to rapidly control the spatial alignment of cells in a 3D matrix while preserving the viability and improving the functionality of patterned cells. Bright-field and confocal microscopy techniques were used to study the linear arrangements of adipose-derived stem cells and their structural preservation over time. The Live/Dead cell assay and alamarBlue^TM^ assay were performed and the alkaline phosphatase enzymatic activity were assessed to investigate the effects of SSAWs on the viability, metabolic activity, and osteogenic differentiation, respectively, of the patterned cells in the hydrogel matrix for up to 14 days. Furthermore, the expression of osteocalcin in the patterned cells in comparison to that in the control cells was imaged using confocal fluorescence microscopy to confirm the potential of this SSAW-based platform for tissue engineering by creating an in vitro biomimetic tissue construct.

## Results and discussion

### Parameter optimization of the SSAW platform using PS particles and MC3T3-E1 cells

#### SSAW working mechanism and device fabrication and working principles

**Fig. 1:**
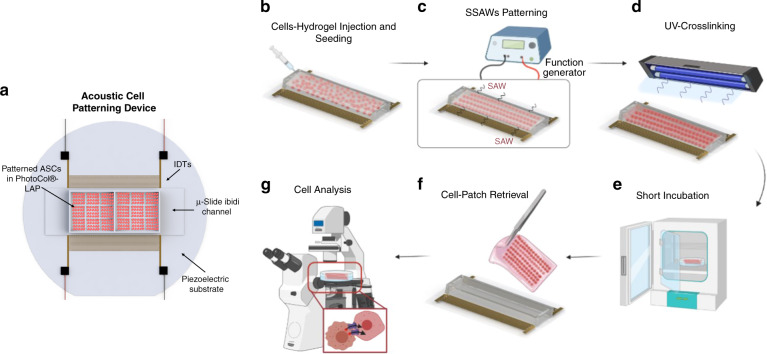
SSAW-patterning platform and workflow of the patterning mechanism and retrievability. **a** Schematic of the SSAW platform. **b** Cell-hydrogel solution is seeded into the platform. **c** SSAW waves are applied to the IDTs via a function generator to create cell patterns. **d** UV light is applied to the patterned cells in the hydrogel to crosslink the scaffold. **e** Brief incubation of the UV-crosslinked cell-hydrogel scaffold is conducted to complete the gelation of the cell-patch. **f** Cell-patch is retrieved from the platform for potential implantation. **g** Cell analysis is performed on Days 1, 7, and 14

Figure 1 illustrates the SSAW-patterning device, which comprises one pair of mirrored IDTs deposited on a LiNbO_3_ substrate. Standing surface acoustic waves (SSAWs) are generated upon the application of a radio frequency (RF) signal to each set of IDTs, where two identical but opposite-traveling waves propagate on the surface of the LiNbO_3_ substrate. The interference between the two traveling SAWs forms a periodic distribution of pressure nodes and antinodes with minimum and maximum pressure amplitudes, respectively. Each set of IDTs consists of 40 pairs of electrodes with a finger thickness (*t*_*IDT*_) and periodic spacing of 75 μm. The wavelength (λ) of the SSAW is 300 μm, defined as twice the pitch (*d*) of the IDT (*d* = *2t*_*IDT*_); thus, the distance between each pressure node is 150 μm, or half a SSAW wavelength. Prior to the optimization of the platform, the theoretical resonant frequency was approximated as $f = c_s/\lambda \approx 13.27MHz$, where c_s_ and λ are the speed of sound in LiNbO_3_ (*3980* *m/s*) and the theoretical SSAW wavelength (*300* $\mu m$), respectively. The working frequency was, however, experimentally optimized and determined to be 13.11 MHz based on both the efficiency of manipulation, *i.e*., how rapidly the cells were guided toward the pressure nodes, and the stability of the pressure lines over time.

The governing force for cell patterning is the acoustic radiation force (ARF), or the time-averaged net force created by pressure fluctuations, and it can be classified into primary and secondary ARFs. Primary acoustic radiation forces emerge from the direct irradiation of acoustic waves on cells, which in turn directs the cell trajectory to the defined pressure nodes, while both secondary radiation forces and drag forces induce and promote cell–cell interactions^25^. The primary ARF (F_r_) acting on suspended, compressible, spherical particles in a liquid medium can be estimated as^26^:

1 $${{{\mathrm{F}}}}_r = - \left( {\frac{{\pi p_0^2V_p\beta _f}}{{2\lambda }}} \right)\phi \left( {\beta ,\rho } \right){{{\mathrm{sin}}}}(\frac{{4\pi x}}{\lambda })$$

2 $$\phi \left( {\beta ,\rho } \right) = \frac{{5\rho _p - 2\rho _f}}{{2\rho _p + \rho _f}} - \frac{{\beta _p}}{{\beta _f}}$$

where p_0_, V_p_, β_f_, βp, ρ_f_, ρ_p_, λ, and x are the acoustic pressure, volume of the particle, compressibility of the fluid, compressibility of the particle, density of the fluid, density of the particle, acoustic wavelength, and distance from a pressure node, respectively. The acoustic contrast factor $\phi ({\upbeta},{\uprho})$ determines the direction of the cells toward a pressure node or antinode if $\phi$ is positive or negative, respectively. Therefore, for particles that are denser than the medium, $\phi ({\upbeta},{\uprho})$ becomes positive, and the primary ARF is directed toward the pressure nodes.

Rayleigh waves, also known as leaky waves, are often used in SSAW devices to pattern cells, as they can efficiently leak into the fluid medium to make contact with the fluid path^27^. When a resonating Rayleigh wave encounters the liquid medium, it generates longitudinal-mode leaky waves, leading to pressure fluctuations within the medium^28,29^. By coupling the channel to the piezoelectric substrate via a drop of water, the waves can efficiently leak and create pressure nodal lines with defined boundaries. Polyimide tape was used in the proposed platform to enclose the SSAW working region and thus improve the intensity of the ARF to pattern cells more efficiently.

For the cell-laden hydrogel constructs, cells suspended in media were mixed with PhotoCol^®^-LAP in a 1:3 ratio. In the proposed design, a commercial ibidi^®^ μ-Slide channel was used due to its excellent optical quality and low acoustic damping. After activating an RF to each of the IDT pairs simultaneously, the cells in PhotoCol^®^-LAP were patterned in parallel lines in less than 1 min. To preserve the pattern of the cell-laden hydrogel constructs, UV light was applied 10 cm from the ibidi^®^ coverlid for <3 min to induce gradual crosslinking while minimizing detrimental effects on the cells. The samples were then placed in an incubator for less than 30 min to complete the gelation process. The patterned cell-laden hydrogel constructs were successfully retrieved using conventional tweezers for potential implantation.

#### Device and experimental parameter optimization

**Fig. 2:**
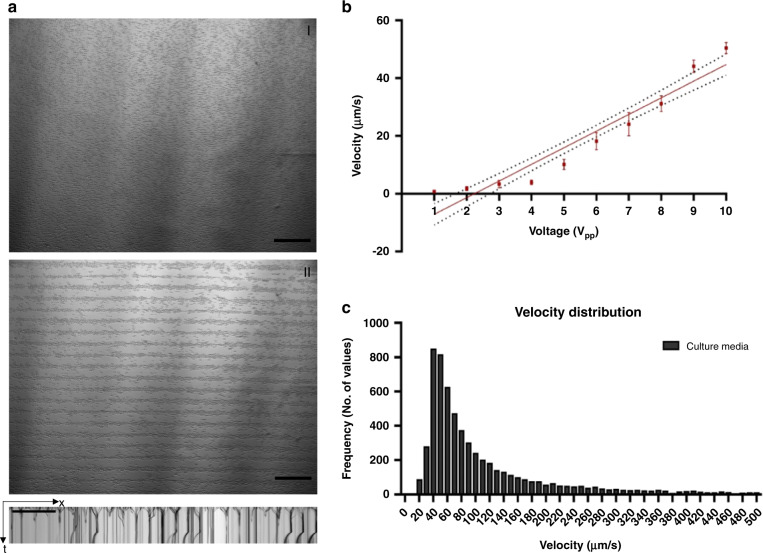
Optimization experimental parameters for cell patterning. **a** Cells prior SSAW activation and after SSAW activation with kymograph image showing nodal line distribution and distance. **b** Velocity vs voltage plot. **c** Velocity distribution of cells in culture media. Scale Bar is 500 μm

For the optimization of the patterning module, parameters including the cell tracking velocity, frequency, and voltage were determined using MC3T3-E1 cells in growth media (alpha-MEM). Figure 2a shows the efficient patterning of SSAWs prior to (i) and after (ii) RF activation, revealing that the proposed SSAW platform is suitable as a high-resolution cell patterning method. The velocity was calculated using the ImageJ TrackMate plugin^30^, where videos were preprocessed to track individual cells at a rate of 50 fps. Ten different voltages were used to capture the displacement of the MC3T3-E1 cells, and as expected, the velocity of manipulation with the working voltage increased linearly (Fig. 2b). Furthermore, Fig. 2c shows the velocity distribution of the MC3T3-E1 cells following a nearly normal distribution, where the majority of cells are moving with an average velocity of ~50 μm/s in αMEM media.

One challenge of using high voltages (>40 V_pp_) that should be taken into account in the design of an SSAW platform is the proportional square increase of the heat generated on the piezoelement substrate. In our platform, the working time in which the function generator is activated to translate electrical potentials into surface acoustic waves is very short (<30 s). Due to this short exposure time, even at high voltages (20 V_pp_ or 0.1 W ~ 20 dBm, for an impedance of 3.7 kΩ at the IDT resonant frequency measured using an Agilent Precision Impedance Analyzer), no hindrance of cell survival is expected. Working with this input voltage and setup (including the coupling layer) did not lead to a significant increase in temperature for the short exposure time. This was confirmed by the absence of crosslinking in PhotoCol^®^, which is a temperature-sensitive biomaterial, during SAW activation without the presence of UV light. Biocompatible and low heat generation in controlled working conditions, for instance, for the manipulation of HeLa cells, was performed using SAWs^31^. This work showed that even after a high-power exposure (23 dBm) for up to 10 min, no significant physiological damages were found to the cell viability and proliferation, where the temperature of the piezoelectric substrate increased less than 3 degrees (25 °C to 27.9 °C). It was also reported by the same group that even at a higher input power of 25 dBm for the manipulation of larger organisms, such as C. elegans, the temperature reached a maximum of 31 °C after 10 min, and thus was not detrimental to cell viability^31^. In another work, U-937 monocyte cells were induced with high-power SAWs (26.7 dBm) and cultured for up to 48 h. These authors reported no detrimental effects on cell viability with a maximum heating of the culture medium of only 0.5 +/− 0.2 °C even in the presence of acoustic streaming^32^. It is important to note that for applications in biological research, a temperature under 40 °C prevents protein denaturation and physiological damage to cells, which is significantly greater than the values that have been reported in the literature using SSAW devices for cell manipulation^33^. Another important design feature of our setup is the use of a nonelastomeric ibidi chamber, which allows us to reduce the absorption of acoustic radiation, resulting in less heat being absorbed and limiting the high-power consumption, improving the efficiency of the device^25^.

Furthermore, parameters including the patterning time, the pressure node distance, the pattern preservation criteria, and cell viability were determined using different media conditions, including αDMEM complete media, serum-free αDMEM, PBS 1X, PhotoCol^®^ 2.0 mg/mL, PhotoCol^®^ 2.5 mg/mL, and PhotoCol^®^ 3.0 mg/mL (Table 1). The time to guide cells toward the pressure nodes was very rapid in αDMEM complete media, serum-free αDMEM, and PBS (1X) (<5 s). However, the cell pattern was not preserved over time, and it was easily disrupted a few seconds after the cessation of the SSAW signal. For the PhotoCol^®^ samples, the time to guide cells was longer but still within seconds (<1 min). One other advantage of PhotoCol^®^-embedded samples is that the cell pattern was maintained over time by rapidly gelling the cell-laden hydrogel using UV light in less than 3 min.

**Table 1:** Parameter selection for SSAW-patterning optimization

Parameter/Condition	Cell density (cells/mL)	Time to guide cells to nodes (s)	Pressure node location (μm)	Cell viability (24 h)	Cell pattern preserved (24 h)
Culture media	1.25 × 10^6^	1.14 ± 0.03	149.56 ± 5.03	93.12 ± 3.23	×
Serum-free media	1.53 × 10^6^	1.94 ± 0.26	156.02 ± 1.21	90.51 ± 2.01	×
PBS 1X	1.23 × 10^6^	0.94 ± 0.26	149.24 ± 3.21	81.09 ± 2.11	×
Collagen 3 mg/mL	9.45 × 10^5^	25.09 ± 1.26	130.04 ± 8.53	93.23 ± 4.34	✓
Collagen 2.5 mg/mL	1.02 × 10^6^	18.94 ± 1.46	140.02 ± 10.41	90.07 ± 3.25	✓
Collagen 2 mg/mL	9.91 × 10^5^	16.45 ± 2.42	152.02 ± 11.21	76.18 ± 4.36	✓

Pressure nodal lines, which serve as an indicator of how reproducible our acoustic patterning method is, were calculated by averaging 100 different pressure nodal lines using ImageJ. The average distance between pressure lines for the MC3T3-E1 cells suspended in growth media was 149.56 ± 5.03 µm. For the serum-free media and PBS 1X solutions, the nodal location corresponded to 156 ± 1.21 µm and 149.24 ± 3.21 µm, respectively. Finally, for the cell-laden PhotoCol^®^ samples, the pressure node distances measured for the 3, 2.5, and 2 mg/mL solutions (initial PhotoCol^®^ concentration) were 130.04 ± 8.53, 140.02 ± 10.41, and 152.02 ± 11.21 µm, respectively. All samples were consistent and matched the theoretical value of 150 μm, or half a SSAW wavelength, suggesting the reproducibility of this method.

Interestingly, a cell viability of >90% was found for both the regular growth media and serum-free media samples, suggesting the gentle nature and stress-free mechanism of SSAWs. However, for samples suspended in PBS 1X, the cells were compromised 24 h after SSAW exposure (<60% cell viability) due to potentially insufficient nutrients in the culture media. In addition, PhotoCol^®^-embedded samples showed lower viability for the 2 mg/mL initial collagen concentration after 24 h, which can be attributed to the higher acetic acid content of the PhotoCol^®^ solution at this lower concentration. In contrast, high viability (>90%) was observed for both 3 and 2.5 mg/mL collagen concentrations after 24 h. Therefore, a collagen concentration of 2.5 mg/mL was found to be not only highly biocompatible but also to have a lower viscosity, reducing the patterning time with higher patterning flexibility.

**Fig. 3:**
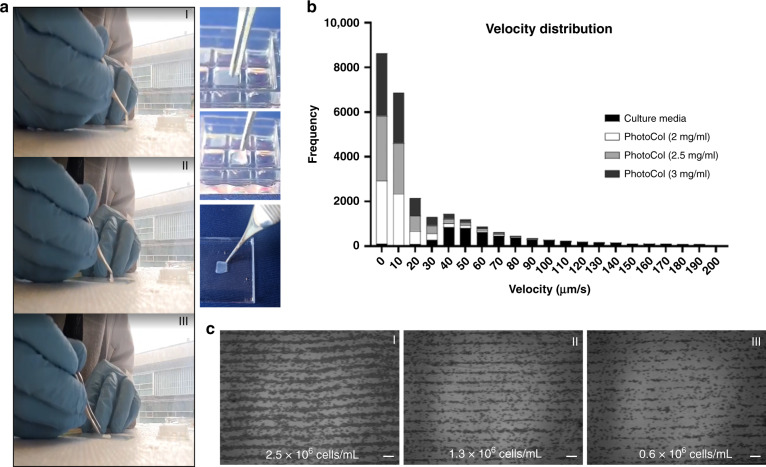
Cell patterning sample optimization **a** Retrievability of the cell-hydrogel construct after 3 min of UV exposure. **b** Velocity distribution of different cell suspension conditions. **c** Cell patterning for various cell densities (I) 2.5 × 106, (II) 1.3 × 106, and (III) 0.6 × 106 cells/mL showing defined patterned lines. Scale bar is 250 μm

Different cell conditions were also used to optimize the acoustic patterning module using culture media. Samples with different cell concentrations (2.5 × 10^6^, 1.3 × 10^6^, and 0.6 × 10^6^ cells/mL) were injected into the ibidi chamber and acoustically patterned. For these three different cell concentrations, the node distances and velocities of manipulation were very similar, indicating that the cell density has a negligible influence on the acoustic patterning using our platform (Fig. 3).

In addition to the initial organization of the cells into the desired pattern, a preservation step is fundamental for establishing cell–cell connections and inducing tissue maturation^16^. To preserve the cell pattern, ECM-based hydrogels are commonly used due to their high biocompatibility and tunable gelation, allowing the maintenance of cellular arrangements over time. For our platform, a UV-sensitive collagen-based matrix (PhotoCol^®^-LAP) was used to form cell-laden hydrogel constructs that were retrievable after gelation. This dose allowed the hydrogel to be crosslinked in less than 3 min. As seen in Fig. 3a, the retrievability of the cell-laden hydrogel constructs from the Mini Well is shown after being exposed to UV light for 3 min. The easy manufacturing steps and easy retrievability using conventional tweezers suggest the great potential of our acoustic patterned cell-laden hydrogel as a regenerative tissue patch for transplantation (see Video).

Furthermore, three different PhotoCol^®^ initial concentrations, 2, 2.5, and 3 mg/mL, were tested and compared to the velocity distribution results obtained using regular growth media. As observed in Fig. 3b, the velocity of MC3T3-E1 cells being acoustically patterned while suspended in growth media was significantly faster due to a lower viscosity than that found for the cell-laden PhotoCol^®^ samples. As expected, the higher the viscosity of the suspension medium is, the longer and less efficient the cell manipulation becomes. The velocity of patterning was reduced to nearly 50% when cells were suspended in PhotoCol^®^ solution (Fig. 3b). However, the average time to guide the cells toward the pressure nodes and induce the formation of linear patterns was still less than 1 min.

### SSAW-patterning of stem cells enhances cell viability, metabolic activity and osteogenic differentiation

#### SSAW-patterning improves ASC cell viability and metabolic activity

**Fig. 4:**
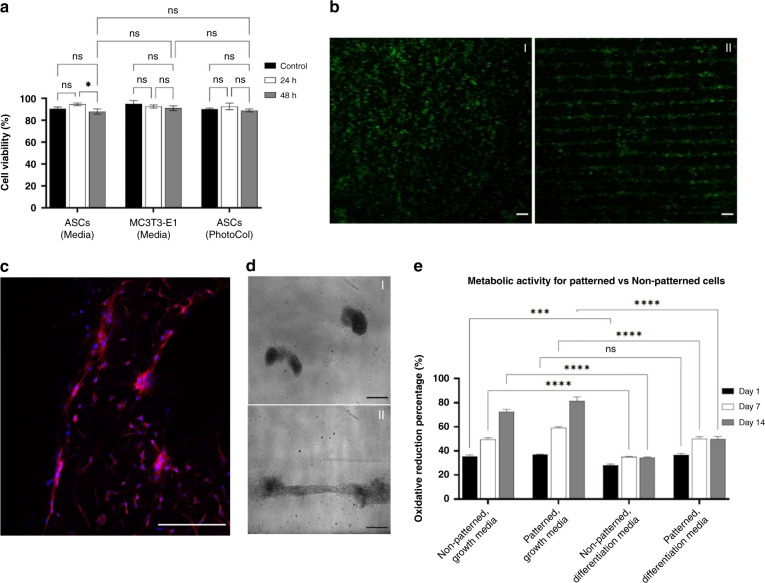
Cell viability and metabolic activity for patterned and non-patterned cells. **a** Cell viability for patterned ASCs and MC3T3-E1 cells with and without PhotoCol^®^ for up to 48 h after SSAW induction (*n* = 3; *p < 0.05; **p < 0.01; ***p < 0.001; ****p < 0.0001). **b** Live/Dead assay showing high cell viability after (I) at time zero and (II) after 48 h of SSAW induction. **c** Acoustically patterned ASCs showing aligned nuclei (Hoechst 33342) and actin (Phalloidin-iFluor 594) fibers after 1 week in growth media. **d** ASC cell–cell structure alignment of non-patterned (I) and patterned (II) cells in PhotoCol^®^ on Day 14. **e** Metabolic activity of acoustically patterned and non-patterned ASCs cultured in growth media and differentiation media. Scale bar is 250 μm

To validate the biocompatibility of our proposed SSAW platform, we evaluated the cell viability over time using a Live/Dead cell kit. ASCs and MC3T3-E1 cells were stained and mixed with media or hydrogel solution before injection into the channel for acoustic patterning. Three test groups were prepared, including ASCs suspended in PhotoCol^®^-LAP (3.3 × 10^4^ cells/mL), ASCs suspended in DMEM/F-12 (5.1 × 10^5^ cells/mL), and MC3T3-E1 cells suspended in αMEM (5.5 × 10^5^ cells/mL). Fluorescent images were taken at 0 h (control), 24 h, and 48 h after SSAW activation. Our results confirm that high cell viability (>90%) was preserved for all groups 48 h after exposure to SSAWs (Fig. 4a). There was no significant difference between the control (0 h), the acoustically patterned MC3T3 cells in αMEM and the ASCs in PhotoCol^®^-LAP at 24 h and 48 h after SSAW induction (*p* < 0.0001, ANOVA two-factor with replication, *n* = 3), which proves the gentle nature of SSAWs, suggesting that they do not affect cell viability. A difference in cell viability was observed between 24 and 48 h for the ASC-DMEM/F12 (media) group; nonetheless, an over 85% cell survival was observed.

One of the challenges of tissue engineering, in addition to the preservation of cell viability, is the maintenance of cellular spatial arrangement over time^34^. By using a methacrylated collagen hydrogel, we could preserve the linear patterns after UV crosslinking, which could also prevent the early degradation of tissues prior to cell differentiation and migration. Figure 4b confirms the preservation of the cell pattern in PhotoCol^®^-LAP 48 h after SSAW activation, showing dense and defined lines. No significant differences in cell viability were observed between the control and acoustically patterned groups.

The effects of UV light on cell-hydrogel photopolymerization and cell viability have been widely reported^3,35–38^. For instance, Lin et al.^37^ showed that endothelial colony-forming cells (ECFCs) and mesenchymal stem cells (MSCs) remained viable (>93%) for up to 120 s and were only negatively affected after 300 s of UV light exposure (7.5 mW/cm^2^). Moreover, this group demonstrated that by increasing the UV exposure time, the spreading capacity of cells inside their hydrogel (GelMA) diminished, which was directly correlated to the degree of GelMA polymerization, as expected. In another study, the effects of UV light on human MSCs photoencapsulated in GelMA were studied. The results showed that upon the induction of UV light (490–510 nm) with an intensity of 20 mW/cm^2^, good cell viability (>75%) was maintained after 4 min of light exposure with a 3% reduction after 10 min^38^. Our results confirmed a similar behavior, as cell viability was preserved for UV-exposed samples exposed <3 min to an intensity of 1 mW/cm^2^ at 10 cm from the source based on the Live/Dead assay. After 48 h of incubation, a high viability of >90% was observed, with no significant differences observed after crosslinking the cells with UV light.

To further investigate the effects of acoustic cell patterning on cell functionality, we quantified the metabolic activity of acoustically patterned ASCs in hydrogel solution (PhotoCol^®^-LAP) and nonpatterned ASCs (control) in PhotoCol^®^-LAP via the alamarBlue™ assay. After sample fabrication, the control and acoustically patterned groups were cultured in growth and differentiation media, and alamarBlue™ assays were performed on Days 1, 7, and 14. As seen in Fig. 4e, both the control and acoustically patterned cell-hydrogel groups cultured in growth media showed continuous proliferation between Days 1 and 14. Acoustically patterned cell-hydrogel constructs cultured in growth media showed 45.53% and 32.03% increases in metabolic activity after Day 7 and Day 14, respectively. Interestingly, a different behavior was observed for the acoustically patterned and control groups cultured in differentiation media after Day 7, where a constant metabolic activity was observed. This null change in the cell-laden hydrogel metabolic activity from Day 7 to Day 14 suggests that the cells started differentiating upon the induction of differentiation media, with a similar behavior for both the control and the acoustically patterned groups.

More interestingly, the acoustically patterned group cultured in growth media showed an enhancement in metabolic activity on Days 7 (17.43%) and 14 (11.44%) compared to that of the control group (*p* < 0.0001, ANOVA two-factor with replication, *n* = 3) (Supplementary Fig. 1A). Similarly, the acoustically patterned group cultured in differentiation media showed a significant increase in metabolic activity compared to that of the control group on Days 1 (28.82%), 7 (35.36%), and 14 (36.43%) (Supplementary Fig. 1B).

In addition, Fig. 4c shows acoustically patterned cells within PhotoCol^®^-LAP, where the cell nucleus and actin fibers are shown to be aligned with a predominant unidirectional orientation after 1 week in culture. Figure 4d shows the cell–cell arrangement and the multicellular structure of the control group and acoustically patterned samples after 14 days in culture using differentiation media. As observed, the cell–cell structural morphology and directional alignment were different between the control (Fig. 4d(I)) and acoustically patterned (Fig. 4d(II)) groups. Nonpatterned cells show a lack of cellular interconnections with a nonspreading cell–cell morphology. Acoustically patterned samples, in contrast, showed the extension of elongated protrusions at the ends of the cell membranes, which suggests that they guide cellular migration and interconnection^39,40^. This geometrical difference could be a result of better cell–cell junctions due to patterning, which suggests the improvement of cell communication due to an upregulation of cadherins, receptors, integrins, and signaling proteins^41^. It has also been previously reported that the extension of pseudopodia and tissue branching increases the strength of intercellular connectivity, which facilitates rapid, long-range communication between cells throughout an organism. Therefore, these qualitative results coupled with the aforementioned metabolic activity data indicate that the cadherin-mediated connections from cell–cell contacts could influence the cell behavior, including the metabolic rate, as previously reported^42^.

#### SSAW-patterning enhances ASC osteogenic differentiation

**Fig. 5:**
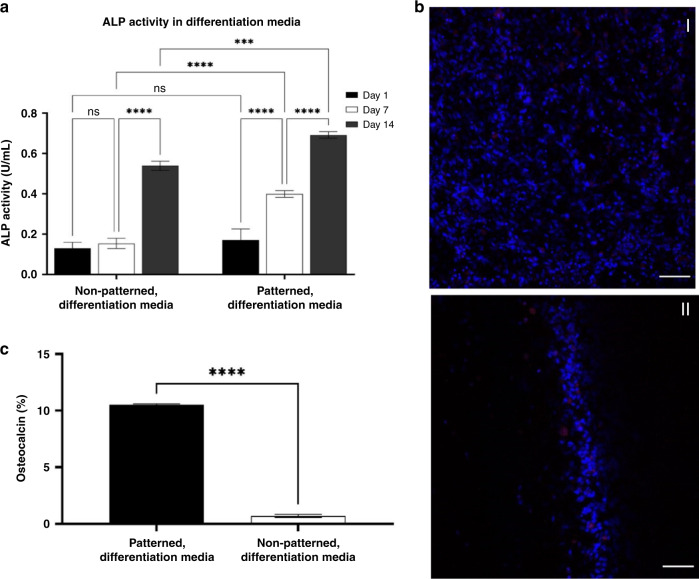
Osteogenic differentiation potential of acoustically patterned cells. **a** ALP activity for non-patterned and patterned cells encapsulated in PhotoCol^®^-LAP hydrogel on Days 1, 7 and 14 (*n* = 3; *p < 0.05; **p < 0.01; ***p < 0.001; ****p < 0.0001). **b** Confocal images of non-patterned (control) cells (I) and acoustically patterned cells (II) with an equal density of 2.5 × 106 cells/mL after 14 days of culture in differentiation media, where osteocalcin is shown in red and nuclei in blue. **c** Osteocalcin percentage difference between the acoustically patterned and non-patterned groups. Confocal images were analyzed using ImageJ to quantify the ratio of the osteocalcin signal area percentage to the total stained area (*n* = 3; ****p < 0.0001). Scale bar is 250 μm

To further validate our SSAW platform and investigate the effects of acoustic patterning on cell functionality, we assessed the differentiation potential of acoustically patterned ASCs in PhotoCol^®^-LAP cultured in differentiation media via ALP activity and osteocalcin signaling. ALP is a membrane-bound enzyme present in osteoblasts and involved in bone mineralization that has been widely used as an early marker of osteogenic differentiation^43,44^. As seen in Fig. 5a, both the acoustically patterned and control groups cultured in differentiation media showed an increase in ALP activity between Days 7 and 14, in correlation with the results obtained for metabolic activity. As ASCs start their differentiation process and change their phenotype, they stop proliferating^45^. Acoustically patterned samples showed an ALP activity enhanced by 80.62% between Days 1 and 7 and by 53.71% between Days 7 and 14. Furthermore, SSAW-based patterning enhances the differentiation potential of ASCs; Fig. 5a confirms an increase in ALP activity of 88.89% at Day 7 and 24.90% at Day 14 for the acoustically patterned samples in comparison to that of the control group. Therefore, acoustically patterned cells experience higher ALP activity due to cell–cell and cell-matrix junctions, which modulate the strength of adhesion and activate mechanosensitive signaling pathways that can influence the cell differentiation process^10^.

Confocal fluorescent images of the acoustically patterned and control groups on Day 14 (Fig. 5b) were in agreement with the ALP activity results. Acoustically patterned ASC-PhotoCol^®^-LAP constructs cultured in differentiation media for up to 14 days had a 10 times higher osteogenic signal (Fig. 5c), quantified as the ratio of osteocalcin area divided by the total stained area. Osteocalcin is a bone-specific protein synthesized by osteoblasts that is typically used as an early marker for osteogenic differentiation in rat mesenchymal stem cells^46^. Therefore, we can observe from Fig. 5b that rat adipose-derived stem cells differentiate into osteoblasts more efficiently for the acoustically patterned group due to improved cell–cell microenvironments that can efficiently coordinate cellular functions^47^, such as cellular differentiation and proliferation for regenerative medicine applications.

Furthermore, our culture platform consisting of major well dimensions of 21.5 × 23.6 × 6.8 mm^3^ and minor well dimensions of 6.1 × 6.8 × 1.3 mm^3^ allowed us to study the effects of cell patterning in 3D. Cells-PhotoCol^®^-LAP solution was evenly divided between the control and acoustically patterned groups at a density of 2.5 × 10^6^ cells/mL. Therefore, Fig. 5b suggests that acoustically patterned cells are aligned throughout the z-direction as ASCs are localized and condensed at the pressure nodal line. Previous work, however, has shown that the cell–cell nodal alignment slightly changes with respect to the height of the channel, where a higher acoustic force is experienced at the surface of a piezoelectric element^3^. We imaged the acoustically patterned cells after 1 week in culture in vertical cross-sections (z-stacks) using confocal microscopy. As shown in Supplementary Fig. 6, the cells aligned into pressure nodes more efficiently when they were closer to the surface of the piezoelectric substrate and dispersed at the surface of the hydrogel, although they still maintained their general pattern. This work thus allowed us to study the structural changes of acoustic patterning and obtain insightful information about the cell–cell behavior, including the enhanced proliferation and differentiation of stem cells.

## Conclusion

In this work, we implemented an SSAW-based contactless cell patterning platform to rapidly fabricate high-resolution cell-hydrogel linear arrangements of adipose-derived mesenchymal stem cells with the capability of being retrieved from the SSAW platform after UV crosslinking. The fast arrangement of the cells in linear patterns with a reproducible linear nodal separation of ~150 μm was successfully shown in <5 s for culture media and <1 min for the PhotoCol^®^-LAP hydrogel. We demonstrated the capability of creating multiple biomimetic tissue patches from aligned adipose-derived stem cells in a UV-crosslinkable PhotoCol^®^-LAP hydrogel that wase easily retrievable from the platform as a proof-of-concept for tissue regeneration. We studied the effects of acoustic cell patterning on enhanced cellular proliferation and osteogenic differentiation, which have never been directly correlated to SSAW-patterning using adipose-derived stem cells. The acoustically patterned cells remained viable with increased metabolic activity and enhanced osteogenic differentiation potential, as revealed by the quantification of alamarBlue™, ALP activity, and osteocalcin signaling for up to 14 days. Cell–cell interconnection was also improved in acoustically patterned cells, which is critical for cell communication, cell migration, and tissue development. Together, these results indicate the great potential of the proposed acoustofluidics platform in constructing tissue patches with induced cell–cell contacts for their application in tissue regeneration and wound healing.

## Materials and methods

### Materials

Methacrylated Type I Collagen with LAP photoinitiator kit (PhotoCol^®^-LAP) was purchased from Advanced Biomatrix (CA, USA). StemXVivo^®^ Osteogenic/Adipogenic Base Media and StemXVivo^®^ Osteogenic Supplement were purchased from R&D Systems (MN, USA). Dulbecco’s modified Eagle’s medium/nutrient mixture F-12 (DMEM/F12), minimum essential medium (MEM-alpha/α-MEM), fetal bovine serum (FBS), penicillin–streptomycin (P/S), antibiotic-antimycotic, formaldehyde solution, and TrypLE™ Express Enzyme were all purchased from Thermo Fisher Scientific (MA, USA). Bovine serum albumin (BSA) and Dulbecco’s phosphate buffered saline 1X (PBS, without calcium chloride and magnesium chloride) were purchased from Sigma Aldrich (MI, USA). alamarBlue™ Cell Viability Reagent, Live/Dead Viability Assay Kit (L3224), and Hoechst 33342 (Trihydrochloride, trihydrate—10 mg/mL solution in water) were purchased from Invitrogen (MA, USA). Alkaline Phosphatase (ALP) Assay Kit (Colorimetric) and the antibody Phalloidin iFluor 594 conjugate (ab176757) were purchased from Abcam (Cambridge, MA). Osteocalcin rabbit polyclonal antibody (primary antibody) was purchased from Proteintech (IL, USA), and Alexa Fluor Plus 647-Goat anti-Rabbit IgG (H+L) High Cross-Absorbed Secondary Antibody was purchased from Thermo Fisher Scientific (MA, USA). A μ-Slide 2-well coculture chamber was purchased from ibidi GMBH (WI, USA). Four-inch 127.86° Y-cut, X-propagating SAW grade lithium niobate wafers were purchased from Precision Micro-Optics (MA, USA). Murine preosteoblasts (MC3T3-E1) were purchased from the American Type Culture Collection (ATCC, Virginia, USA). Subcutaneous adipose tissue harvested by Dr. Hadil Al-Jallad and Antoine Karoichan from two 3- to 4-month-old Wistar rats was provided by the Research Institute of McGill University’s Health Centre’s (RI-MUHC) animal facility, as described previously^45^.

### Fabrication of the SSAW platform

The SAW device was fabricated using standard soft lithography, e-beam evaporation, and lift-off methods. A 7 μm-thick photoresist layer (S1813, MicroChem, TX, USA) was spin-coated on a 500 μm thick double-sided polished 127.86° LiNbO_3_ piezoelectric wafer. A double layer of titanium and gold (Ti/Au 0.1/1.0kȦ 10 –100 nm) was deposited on the substrate using an e-beam evaporator (bjd1800, Airco Temescal, CA, USA). Next, the substrate was submerged in a developer (MF319, Microposit) at 70° under sonication to remove the undesired coating and form a pair of interdigitated transducers (IDTs). The design of the IDT comprises finger and spacing gaps of 75 μm (or a period of 300 μm) for a total of 40 electrode pairs. A commercial μ-slide well coculture channel with major well dimensions of 21.5 × 23.6 × 6.8 mm^3^ (w × l × h) and minor well dimensions of 6.1 × 6.8 × 1.3 mm^3^ (w × l × h) was used to chamber the cells by coupling them to the piezoelectric substrate using a drop of water. Polyimide tape was used to delimit the SSAW working area and eliminate any interactions of SSAWs with the outer substrate region.

### Cell culture

ASCs were seeded in 0.1% gelatin-coated T75 flasks and cultured in Dulbecco’s modified Eagle medium (DMEM/F-12) supplemented with 10% fetal bovine serum (FBS), 1% penicillin/streptomycin (P/S), and 1% antibiotic-antimycotic (AA) growth medium. At 70-80% confluency, the cells were trypsinized using TrypLE Express Enzyme (1X), where cells were used before passage 7 for all experiments. MC3T3-E1 cells were cultured in alpha-DMEM supplemented with 10% fetal bovine serum (FBS) and 1% penicillin/streptomycin (P/S, GIBCO, USA) in T-75 flasks. Before the experiments, the cells were detached into flasks using 0.25% trypsin-EDTA. The cell density was measured using a Countess II FL Cell Counter (Invitrogen, Thermo Fisher Scientific, MA, USA).

### Experimental setup for the cell patterning platform

For the optimization of the cell patterning parameters, including the amplitude, working frequency, velocity, and cell density, both 9.9 μm polystyrene (PS) particles and MC3T3-E1 cells were used. The PS particles and MC3T3-E1 cells were suspended in deionized water and growth media (αDMEM), respectively. At passages 20-30, MC3T3-E1 cells were retrieved using 0.25% trypsin-EDTA. For Live/Dead, alamarBlue™, and ALP activity quantification, ASCs were used. At passage 4, ASCs were retrieved using TrypLE 1X for a final cell concentration of 500,000 cells in 200 μL DMEM/F-12 media.

PhotoCol^®^-LAP was prepared following the manufacturer’s protocol. Briefly, methacrylated type I collagen (PhotoCol^®^), diluted in acetic acid to a concentration of 3 mg/mL, was neutralized and mixed with 2% LAP (photoinitiator, 17 mg/mL in PBS 1X). ASCs cells in 200 μL media were further diluted with the PhotoCol^®^-LAP solution at a ratio of 1:3.

Cell-laden hydrogel constructs without SSAW activation, i.e., the no-cell-patterning (control) group, were compared to cell-laden hydrogel constructs subjected to SSAWs, i.e., acoustic cell patterning. For the control group, cells-hydrogel solution (50 μL) was evenly injected into the minor wells of an ibidi μ-Slide channel with a random cell distribution. No acoustic force was applied.

Conversely, for the acoustically patterned group, the same density of cell-hydrogel solution was injected into the minor wells of an ibidi μ-Slide channel. Two pieces of polyimide tape (3 M, Maplewood, MN, USA) were placed on the LiNbO_3_ substrate to delimit the SSAW working region and to create a space for the coupling liquid between the piezoelectric substrate and the channel. The ibidi channel was then coupled to the piezoelectric substrate using a drop of water. One independent and controllable AC radiofrequency (RF) signal was generated using a function generator (AFG3022C, Tektronix, OR, USA) connected to an amplifier (25A250A, Amplifier Research, PA, USA). The input voltage was set between 10-40 Vpp, and the working frequencies varied between 12.5-13.11 MHz. The cell movement was tracked and recorded using a QImaging camera (Retiga 2000R, ARI, USA) connected to an inverted microscope (TE2000U, Nikon Eclipse, MA, USA).

Once the patterns were formed (<1 min), the function generator was turned off, and both control and acoustically patterned cell-hydrogel constructs were exposed to UV light (405 nm, ~1 mW/cm^2^) at a distance of 10 cm from the surface of the ibidi^®^ coverlid for <3 min to induce gradual crosslinking. The photocrosslinked constructs were then placed in a 37 °C incubator for 30 min. After a brief incubation period, 1 mL DMEM/F-12 media (growth media) or StemXVivo^®^ Osteogenic Base Media (differentiation media) was added to each of the 2 major wells. The growth or differentiation media was changed every 3-4 days for up to 14 days.

### alamarBlue live/dead cell evaluation

For this set of experiments, three test groups were analyzed: ASCs suspended in PhotoCol^®^-LAP, ASCs suspended in DMEM/F12, and MC3T3-E1 cells suspended in αMEM. Briefly, 50 µL of ASCs-PhotoCol^®^-LAP (3.3 × 10^4^ cells/mL), ASCs-DMEM/F12 (5.1 × 10^5^ cells/mL), or MC3T3-E1-αMEM (5.5 × 10^5^ cells/mL) was injected into the mini-wells of the ibidi channel. The channel was coupled to the LiNbO_3_ wafer, and an RF signal of 13.11 MHz at 40 Vpp was exerted to induce SSAWs for <1 min. The SSAWs were turned off, and fluorescent images were captured after 0 h, 24 h, and 48 h. Living cells were labeled with green fluorescence using calcein AM, while dead cells were labeled with red fluorescence using ethidium homodimer-III (Live/Dead viability kit) following the manufacturer’s protocol. The cellular viability was calculated as the percentage ratio of green-fluorescent cells (Live) divided by the total fluorescent cell area (both Live and Dead) using ImageJ. A replicate of three samples was used for the analysis.

### Metabolic activity of encapsulated ASCs

To study the effects of acoustic cell patterning on cell metabolic activity, we compared both nonpatterned (control) and acoustically patterned cell-hydrogel constructs. Both groups were cultured in growth media (DMEM/F12) and differentiation media (StemXVivo^®^ Osteogenic Base Media) to further investigate the impact of acoustic cell patterning on osteogenic differentiation. The ^TM^ assay was used to measure the metabolic activity of ASCs-PhotoCol^®^-LAP constructs following the manufacturer’s protocol. On Days 1, 7, and 14, the media was removed, and the cell-hydrogel constructs were washed twice with PBS. The samples were then covered with a 10% alamarBlue^TM^ reagent solution in complete growth medium and incubated for 4 h at 37 °C and 5% CO_2_. After incubation, the supernatant was collected in a 96-well plate, and the absorbance was measured at wavelengths of 570 nm (excitation) and 600 nm (emission) using spectrophotometry (SpectraMax i3, SoftMax Pro 6.3, Molecular Devices, CA, USA). The samples were prepared in triplicate, and the absorbance of each replicate was also measured in triplicate. The measurements were averaged and used to calculate the percent reduction of alamarBlue^TM^ using following the equation:

$$\frac{{(\varepsilon {{{\mathrm{ox}}}})600 \times {{{\mathrm{A}}}}570 - (\varepsilon {{{\mathrm{ox}}}})570 \times {{{\mathrm{A}}}}600}}{{(\varepsilon {{{\mathrm{red}}}})570 \times {{{\mathrm{A}}}}^\circ 600 - (\varepsilon {{{\mathrm{red}}}})600 \times {{{\mathrm{A}}}}570}} \times 100$$

where *A* is the absorbance value of the experimental sample, *A*° is the absorbance value of the control, and (ε*ox*)570 = 80,586, (ε*ox*)600 = 117,216, (ε*red*)570 = 155,677, and (ε*red*)600 = 14,652.

### Alkaline phosphatase (ALP) activity for osteogenic differentiation

The supernatant of acoustically patterned and control ASC-PhotoCol^®^-LAP constructs was collected in 1.5 mL microcentrifuge tubes and snap-frozen in liquid nitrogen on Days 1, 7, and 14. Samples were then stored at −80 °C until the assay was performed. The ALP activity was quantified using a commercial ALP kit (colorimetric) following the manufacturer’s protocol.

### Immunofluorescence

On Day 14, the supernatant was removed from the ibidi channel, and the cell-hydrogel constructs were washed twice with 1X PBS. Cells were fixed in 4% paraformaldehyde for 30 min, permeabilized with 0.1% Triton-X in PBS for 10 min and blocked with 2% BSA solution in PBS for 30 min. After blocking, the samples were incubated with osteocalcin rabbit polyclonal antibody (1:100) in 0.2% BSA solution overnight at 4 °C. The next day, the samples were incubated with the secondary antibody Alexa Fluor Plus 647-Goat anti-Rabbit IgG at a 1:500 dilution in 0.2% BSA for 1 h at room temperature. The cellular nuclei and actin were stained with Hoechst 33342 (1:1000) and phalloidin iFluor 594 (1:1000), respectively, in 0.2% BSA solution for 1 h at room temperature. Samples were stored at 4 °C until imaging. Fluorescent images were captured using confocal laser scanning microscopy (LSM 710, Zeiss AxioObserver).

### TrackMate analysis

The TrackMate plugin was used to evaluate the cell-patterning velocity. A Laplacian of Gaussian (LoG) filter was used with Blob diameter and intensity threshold inputs. The bright field videos were processed by first inverting the contrast using the inverted LUT from the lookup tables and applying a variance filter to give a fluorescent-like image appearance to detect individual cells. The Blob diameter was estimated as the cell diameter (15 μm). These spots were then linked into trajectories using the Linear Assignment Problem (LAP) tracker. The velocity was then calculated from the spot data for 50 fps using the following equation:

$$v = \frac{{\sqrt {\left( {x^2 + y^2} \right)} }}{{\left( {\frac{{frame}}{{50fps}}} \right)}}$$

### Statistical analysis

Three independent replicates (*n* = 3) of each group were performed for each experiment. Data are presented as the mean ± SD. Two-way analysis of variance (ANOVA) with Tukey’s test was used to assess the statistical significance of the data at 95% confidence. Graphs were prepared using GraphPad Prism.

## Supplementary information

Video of self-standing patch

Supplemental Material

## Data Availability

The data that support the findings of this study are available from the corresponding authors upon reasonable request.
