# Supplementary material for: Enhancing metabolic activity and differentiation potential in adipose mesenchymal stem cells via high-resolution surface-acoustic-wave contactless patterning
Source: Microsyst Nanoeng. 2022 Jul 12;8:79. doi: 10.1038/s41378-022-00415-w (PMC9276743; doi:10.1038/s41378-022-00415-w)
Supplement: Supplementary file 2 — Supplemental Material [file 41378_2022_415_MOESM2_ESM.docx]

**Supplementary Information**

**Enhancing Metabolic Activity and Differentiation Potential in Adipose Mesenchymal Stem Cells via High-Resolution Surface Acoustic Waves Contactless Patterning**

*Karina Martinez Villegas,^1^ Reza Rasouli,^1^ and Maryam Tabrizian* ^1, 2^*

*^1^ Department of Biological and Biomedical Engineering, ^2^ Faculty of Dental Medicine and Oral Health Sciences, McGill University, Montreal, QC, Canada*

***Corresponding author:**

*Dr. Maryam Tabrizian*

3775 Rue University, Montreal, QC. H3A 2B4

Phone: (+1) 514-398-8129

Fax: (+1) 514-398-7461

Email: [maryam.tabrizian@mcgill.ca](mailto:maryam.tabrizian@mcgill.ca)

Other authors information:

*Karina Martinez Villegas*

E-mail: [karina.martinezvillegas@mail.mcgill.ca](mailto:karina.martinezvillegas@mail.mcgill.ca)

*Reza Rasouli*

E-mail: [reza.rasouli@mail.mcgill.ca](mailto:reza.rasouli@mail.mcgill.ca)


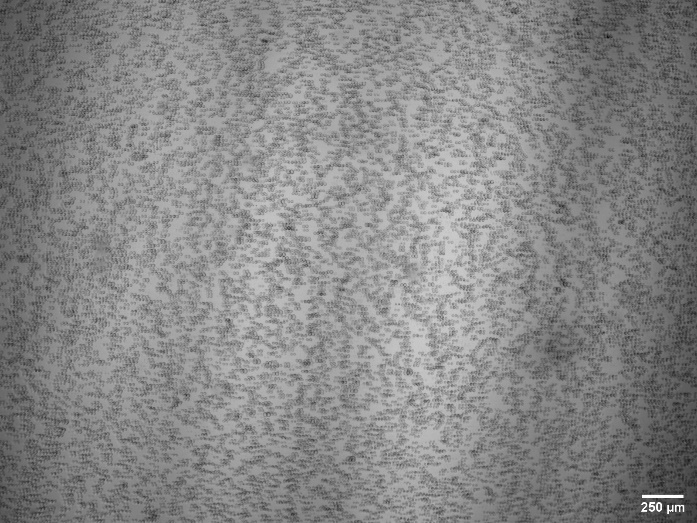

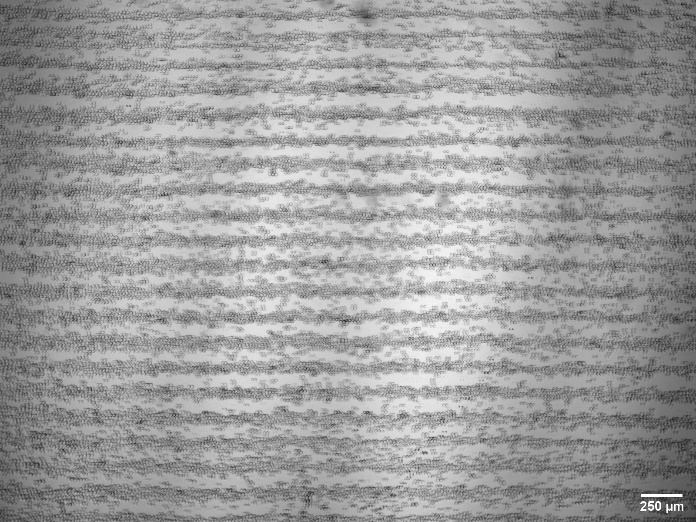


**B**

**A**

Supplementary Figure 1: Brightfield images of high-resolution standing surface acoustic wave patterning of MC3T3-E1 cells showing 20 well-defined pressure nodal lines. A) Non-patterned cells showing random distribution of cells. B) Acoustically patterned cells showing defined lines of high-density cells (1.5 x10^6^ cells/mL) in growth media. Scale bar is 250µm.


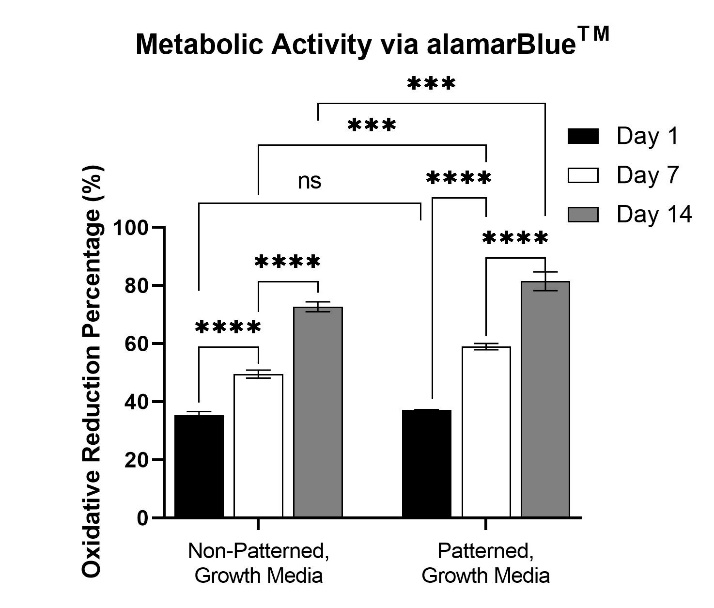

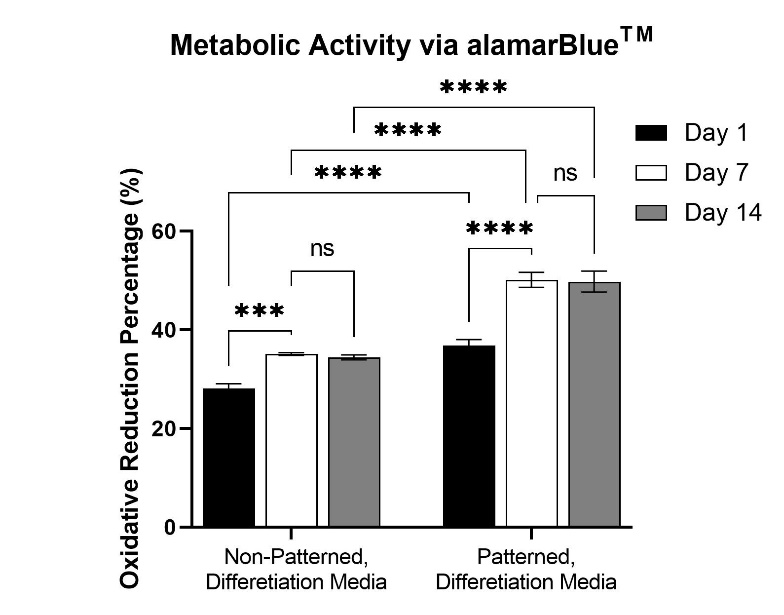


**B**

**A**

Supplementary Figure 2: Metabolic activity as an indicator of cell proliferation of acoustically patterned and non-patterned (control) cells in PhotoCol®-LAP hydrogel cultured in growth and differentiation media. A) Acoustically patterned cells in PhotoCol®-LAP cultured in growth media show a significantly different increase in metabolic activity compared to control samples for days 7 and 14 (n=3; ****: p < 0.001). B) Metabolic activity of patterned and control samples, where acoustically patterned samples have an increase in showing a signf increase for acoustically patterned cells in PhotoCol®-LAP cultured in differentiation media with statistical significance for days 7 and 14 (n=3; ****: p < 0.0001).


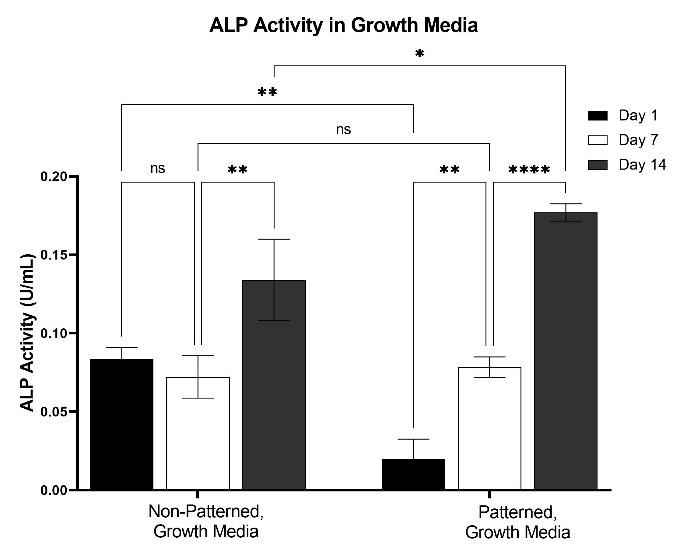

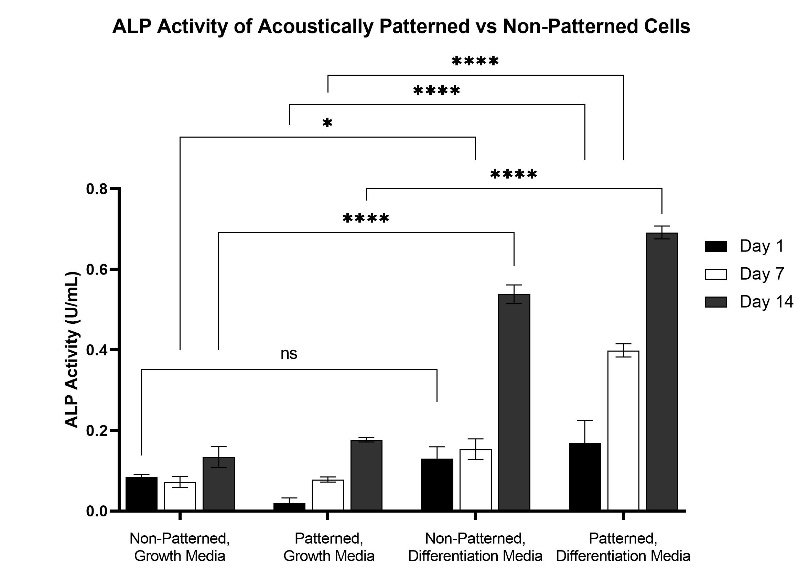


**B**

**A**

Supplementary Figure 3: Alkaline Phosphatase Activity of acoustically patterned cells in PhotoCol®-LAP hydrogel cultured in growth and differentiation media. A) Alkaline Phosphatase activity showing slight upregulation of ALP activity at day 14 of acoustically patterned samples cultured in growth media with statistically significance for days 1 and 14 (n=3; *: p < 0.05; **: p < 0.01; ***: p < 0.001;****: p < 0.0001). B) Alkaline Phosphatase Activity of all groups combined showing significant difference between growth and differentiation media with an increase in ALP for cell-laden hydrogels cultured in differentiation media after days 1, 7, and 14 (n=3; *: p < 0.05; **: p < 0.01; ***: p < 0.001;****: p < 0.0001).


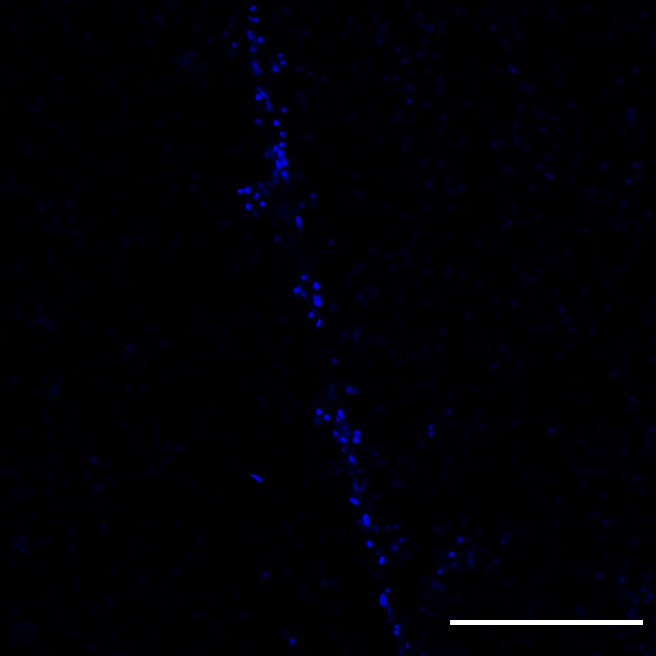

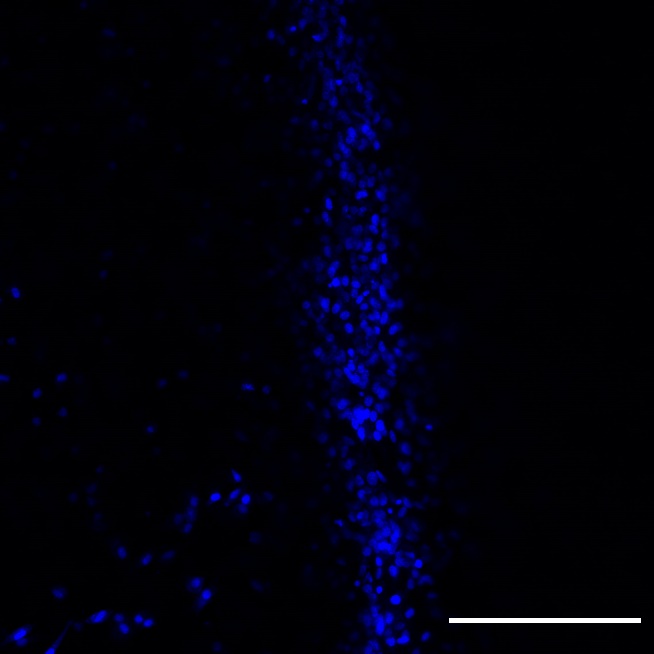


**B**

**A**

Supplementary Figure 4: Acoustically patterned adipose-derived stem cells (ASCs) showing nucleus (Hoechst 33342) in single pressure node cultured in growth media after (a) one week of culture and (b) two weeks of culture. Scale bar is 250μm.


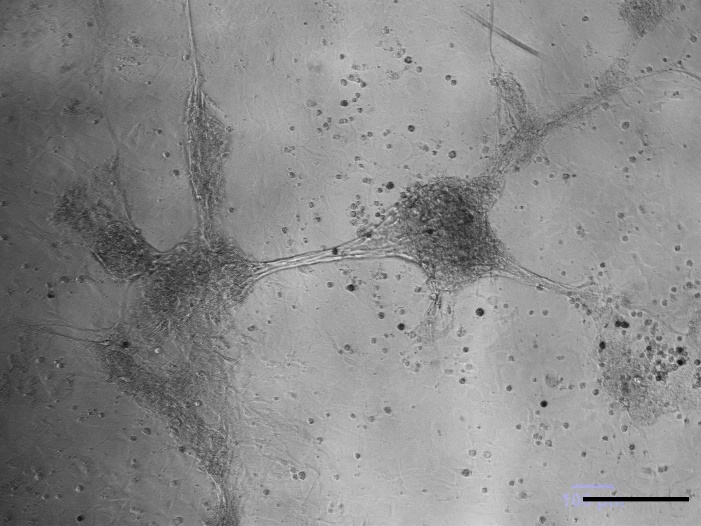

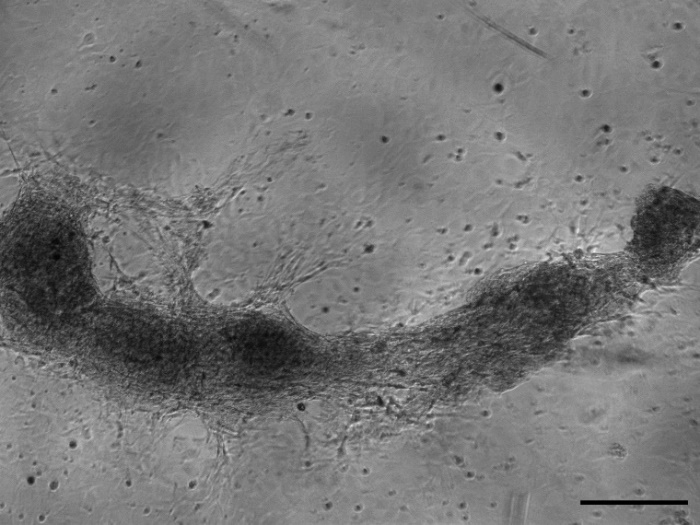


**B**

**A**

Supplementary Figure 5: Cell morphology for control and acoustically patterned adipose-derived stem cells in PhotoCol®-LAP hydrogel after 14 days cultured in differentiation media. A) Random distribution of ASCs led to poor cellular interconnections with few cell membrane protrusions. B) Acoustically patterned cells showed high-levels of cellular interconnections with a dense spreading-like morphology suggesting strong cell-cell interconnections. Scale bar is 250µm.


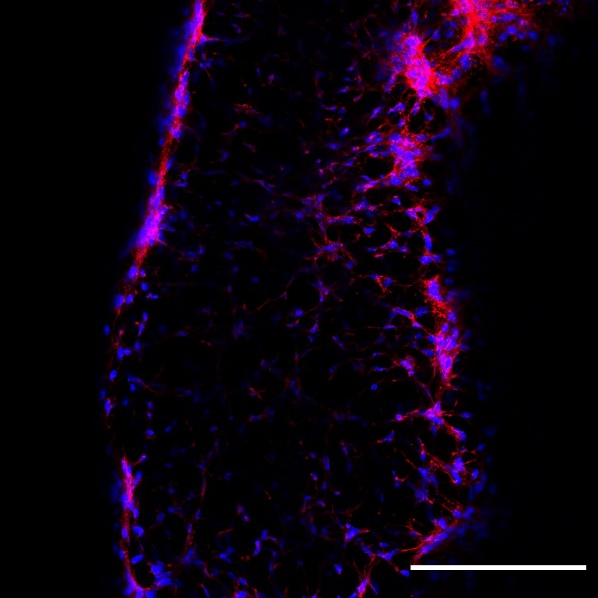

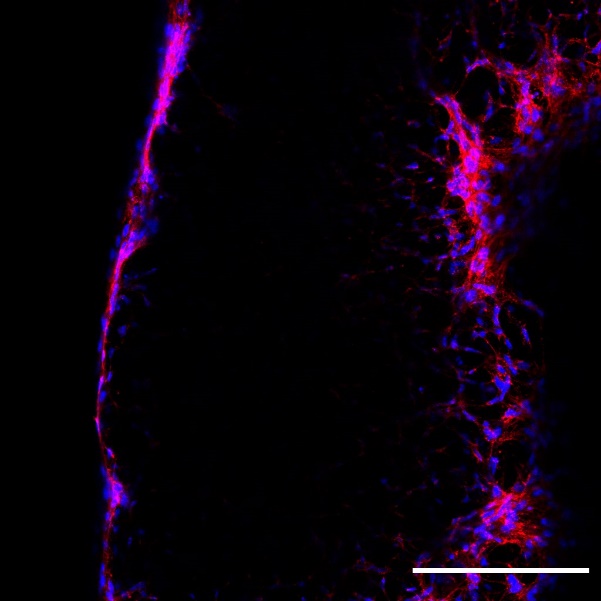

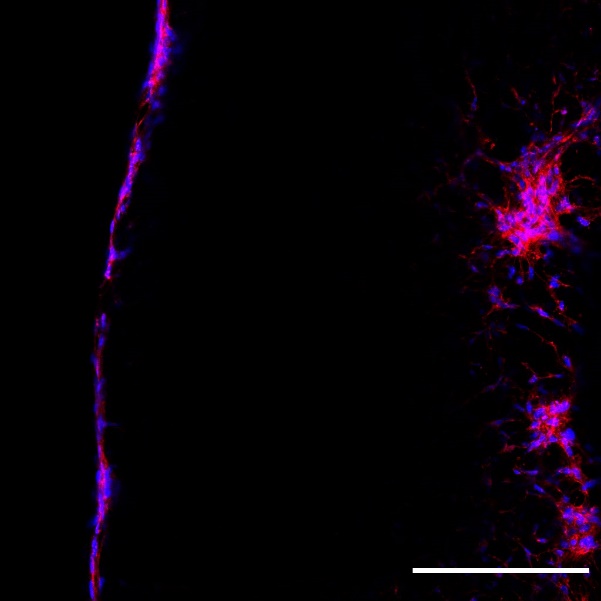

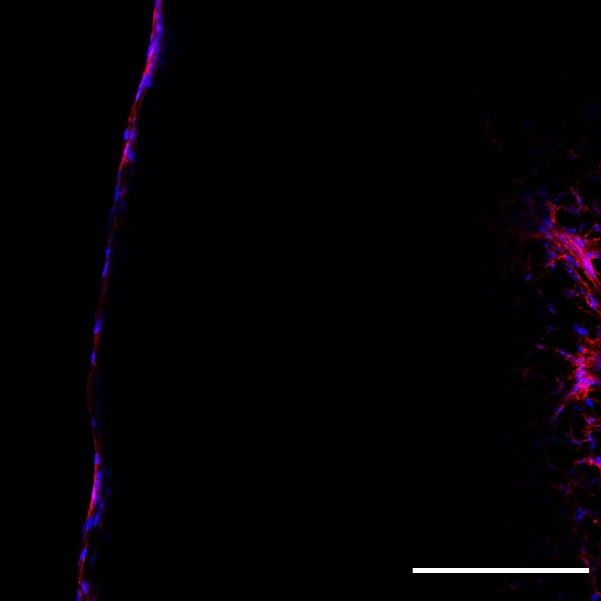


**D**

**C**

**B**

**A**

Supplementary Figure 6: Z-stack projection of acoustically patterned adipose-derived stem cells (ASCs) showing aligned nucleus (Hoechst 33342) and actin fibers (Phalloidin-iFluor 594) cultured in growth media after one week of culture from closer point to the piezoelectric substrate (A) to furthest point from the substrate (D). Scale bar is 250μm.
